# Impact of IoT on Achieving Smart Primary Healthcare Building Facilities in Gauteng, South Africa

**DOI:** 10.3390/ijerph191811147

**Published:** 2022-09-06

**Authors:** Nuru Gambo, Innocent Musonda

**Affiliations:** 1 Centre for Applied Research and Innovation in the Built Environment, University of Johannesburg, Johannesburg 2006, South Africa; 2 Department of Quantity Surveying, Faculty of Environmental Technology, Abubakar Tafawa Balewa University Bauchi, Bauchi 740272, Nigeria

**Keywords:** primary healthcare, building services, IoT, construction, smart, South Africa

## Abstract

Processes and services undertaken in smart primary healthcare building facilities capture operational data through advanced monitoring and enable experts to use these building facilities for efficient healthcare service delivery. This study assessed the impact of Internet of Things (IoT) services on achieving efficient primary healthcare in the rural areas of South Africa. The study identified three (3) basic constructs of IoT services. They include IoT location recognition and tracking services, the application of the IoT high-speed communication network-based services, and the application of IoT-based services. The study is quantitative, and a questionnaire was used to collect data from the project managers and healthcare practitioners working with the primary healthcare agency in South Africa. The study found a variable degree of impact between the three (3) IoT constructs and the successful development of primary healthcare building facility services in South Africa. The study recommends adopting IoT essential services for achieving efficient primary healthcare services in the rural areas of South Africa and other developing countries facing similar primary healthcare delivery challenges.

## 1. Introduction

With recent advances in information technology (IT), smart healthcare buildings have gradually come to the fore. Smart healthcare building facilities use a new generation of technologies such as the Internet of Things (IoT). These technologies are capable of addressing challenges faced by traditional medical service delivery, making them more efficient, convenient, and personalized. The concept of a smart healthcare building facility services entails the application of smart technologies to support healthcare services and introduce smart healthcare [1].

Most studies on smart healthcare have focused on the benefits, barriers, and determinants of adopting information and communication technology (ICT)-based solutions within healthcare building facilities. However, understanding the impact of essential IoT services regarding the achievement of efficient primary healthcare remains problematic considering the issues associated with traditional healthcare service delivery in developing countries. Health facilities in developing countries are characterised by dilapidated building facilities and the poor coordination of healthcare services, among other challenges [2].

Most studies tend to adopt methods of data collection and analysis that are barely credible. Jaafar et al. [3] noted that healthcare building facility services are globally charged with multiple inherent risks. Similarly, Yuan et al., (2022) [1] noted that healthcare buildings have no capacity, especially in developing countries, and are faced with the problems of capturing data for indoor environments, air quality and thermal comfort.

A study conducted on healthcare organization performance factors in private clinics of Addis Ababa in Ethiopia by Wassie et al. [2] found that 61.2% of the clinics experienced poor healthcare waste management services. Another study found that 56.8% of the clinics had poor waste segregation services, and 55.0% of the clinics had poor waste collection methods. In addition, this study also found that clinics’ handling of waste was poor: waste transportation methods, storage services, treatment methods and disposal systems were all inadequate.

However, there are now opportunities to change the status quo. In the UK, Wanigarathna et al. [4] noted that building information modelling (BIM) and the IoT have the potential to enable better and well-versed decision-making concerning the built asset. Built asset management (BAM) is now achieved through the integration of a variety of data that are related to the physical condition of the built facility. However, the significant shortcomings of using BIM for data collection are its incompatibility, i.e., it is not commonly and universally used among construction professionals, and there are legal issues with its software applications. Equally, the cost of the software requires considerable investment.

In Philippines, Dela Cruz and Tolentino [5] stated that the problems associated with poor information gathering in the management of public healthcare facilities originated mainly because of poor financial resources allocation, improper deployment of the right materials, and technologies such as IoT. Additionally, the problem of the planning process is another major factor influencing the effective management of healthcare building facilities [1].

Similarly, Jia et al. [6] examined the process of implementing IoT for the management of healthcare building facilities. This study aimed to enhance the management process of healthcare construction and operation and the impact of the built facility on services, efficient functionalities, and enhancing sustainability. However, the study did not look at assessing the extent to which technology enabled data gathering and analysis for primary healthcare facilities in developing countries.

Moreover, Zhao and Jiang [7] introduced Insect Intelligent Building (I^2^B), which operates based on IoT. The main features of the technologies were developed from agent models. The I^2^B used space units and control devices that were developed from the model. This connects between the devices and the surrounding spaces, i.e., the control devices (insect) for each space unit or each control device have six data ports (legs). The control devices communicate with each other via the data ports based on the developed model. The network performs various operations within the building facility and computes tasks that run on smart controllers, depending on the associations among neighbouring controllers to achieve the desired effects of the commands. The main shortcomings of this system are the problems associated with the standard description of the device and the unit space. Then, there is the issue of the systematic process that changes the facility control programs into parallel computing tasks, which are successively used on the smart nodes and in the communication procedures.

Therefore, the current paper assesses the impacts of IoT on achieving smart primary healthcare building facility services through the assessment of technologies suitable for achieving smart primary healthcare building facilities with a view to enhance healthcare delivery in the rural areas of South Africa.

The following objectives of the paper are:

1. To explore the major IoT technologies fostering smart primary healthcare building facility services;
2. To assess the impact of the IoT technology services on the successful development smart primary healthcare buildings.

### 1.1. Literature Review

In this section, a detailed discussion of the smart technologies employed in primary healthcare facilities is presented. The literature review was carried out on smart building facilities, technologies influencing smart healthcare building facilities and IoT technologies fostering smart healthcare buildings.

### 1.2. Smart Building Facilities 

Globally, the smart building market is rapidly expanding, largely driven by the IoT and new types of technologies. The healthcare building facilities remain an essential component in improving healthcare services. A smart healthcare building facility provides a healthier energy efficiency and controls the safety aspects of the healthcare facility, including the framework for the comfortability of residents and enhanced quality of life and serviceability [8]. Definitions of smart building facilities were proposed and mainly focused on energy features linked to the concept of a “smart grid”. A healthcare building facility that incorporates smart management systems and huge storage of data and analytics that facilitates easy management of energy in the facility. The electrical facilities on the grid that determine the pattern and behaviours within the facility are regarded as a smart/intelligent building facility system. Consequently, the management process of such devices is smart/intelligent via the adoption of IoT technologies [9].

Generically, a smart healthcare facility comprises of three (3) levels, including: The level of the infrastructural data inputs, which embodies all sources of the data collected by the devices, such as consumed energy, level of humidity, indoor/outdoor temperatures, safety alarm activation and deactivation and so on. Then, the level of the facility system signifies the fundamental of the smart system, because this permits the gathering, processing, assembling and storage of the information in a Not Only Structured Query Language (NoSQL) database system. Accordingly, the system permits the utilization of the collected data for the extraction of knowledge by data mining systems and the process of automatic learning through artificial intelligence (AI) algorithms [10].

In the age of IoT, Khan and Salah [11] described the basic features of a smart healthcare building facility as any healthcare building with interoperable building facilities and a mobile integrated solution, while Wassie et al. [2] added that smart healthcare building facilities should have features such as the digitisation of information and established unified systems of communication [9]. Smart building facilities are linked online. Greater attention is also being paid to integrated building automation in the renovation and construction of new buildings. Buildings for which smart technology is applied are called smart buildings.

Moreover, the acronym “SMART”, meaning “Self-Monitoring Analysis and Reporting Technology”, is a technology that provides reasoning alertness to objects, by using innovative technologies such as IoT, artificial intelligence (AI), machine learning (ML) and an extensive analysis of the collected data, providing an intellectual understanding of the facilities that were earlier regarded as useless [12]. Smart technologies are networks of devices that use IoT sensor devices and software and are connected online. This system brings static physical facilities to life. The highly valuable devices are sustainable, mountable, and automated.

### 1.3. Technologies Influencing Smart Healthcare Building Facilities (SMAHEAL)

IoT is a significant technological network of devices that uses the internet connectivity of sensor devices and software that animate static physical facilities [13]. Baqer et al. [14] described smart appliances within healthcare building facilities as critical IoT technologies that can support the achievement of primary healthcare building facilities in developing countries. Smart connected devices in primary healthcare building facilities can be remotely controlled with long-term evolution (LTE), Bluetooth, cellular connectivity and Wireless Fidelity (Wi-Fi) [1].

According to Kwon et al. [9], smart primary healthcare building facilities are commonly categorized into three (3) constructs: Services based on location recognition and tracking technology, which evaluates and monitors the location of any data in the facility based on short-range communication technology; the high-speed communication network-based services, which is an installed wireless communication technology; and the construct of IoT-based services, which are used to link IoT devices embedded with sensors and communication functions to the internet. 

### 1.4. IoT Location Recognition and Tracking Services (IoT-LORE)

IoT facility services are usually achieved by measuring and monitoring the location-based information of any functional facility within a healthcare building space, with the aid of location recognition and tracking technology constructed on short-range communication technology [9,15]. The major technologies associated with IoT in the management of healthcare building facilities are Bluetooth technologies, beacons technologies, Wi-Fi technologies, Zigbee technologies, radio frequency identification (RFID) and global positioning system (GPS) technologies, assisted global positioning system (A-GPS) technologies, barcodes and quick response (QR) codes technologies and the ultra-wideband and communication technologies, e.g., 5th Generation (5G) technology [9]. With the introduction of a tracking system within the healthcare building facility for real-time assets in healthcare services using IoT devices, medical institutions can enhance the effectiveness of logistics management, which is related to healthcare building facilities, and hence improves the workflows of medical staff in the facility [16]. A smart infusion pump involving RFID was first introduced in the US healthcare system, where this technology enhanced the productivity and effectiveness of healthcare building facility through the reduction of about 80% in the time taken by the medical staff to locate any facility within the healthcare centre. The location system utilised real-time location monitoring [9].

### 1.5. IoT High-Speed Communication Network-Based Services (IoT-HISB)

IoT-HISB, such as 5G networks and Wi-Fi 6 networks, can help deliver healthcare building facilities with internet services. If such a technology is utilised, the shortcomings of real data collection and analysis processes can be eliminated. These systems of communication are constructed on wireless communication technology, such as the Wi-Fi 6 technology, as one of the highspeed communication network services. This is mainly used in healthcare buildings where there is high traffic involving regular changes in the environment [17,18]. The introduction of orthogonal frequency-division multiple access (OFDMA) technologies, which combine multiple users with different times and requirements to simultaneously gain access to a single-access point in a healthcare building, reduces the transmission waiting time.

The application of Wi-Fi 6 technologies helps with the accurate analysis of the records of patients within the building facility. These real-time data also help to improve treatment outcomes through the precise administration of medication. This is achieved by an objective decision-making procedure according to the accurate and current patient database [2]. The technology of Wi-Fi 6 aids medical devices, such as infusion pumps with adjustable data to transfer times and reduce usage overlap, improving the efficiency of facility operation and maintenance. This is also achieved through OFDMA by allowing about thirty (30) different facility devices to use the same infusion pump and channel without changing orders (Yan, 2019 [7,19]).

### 1.6. IoT-Based Services (IoT-BAS)

IoT-BAS is a technology that links different facilities entrenched in sensor devices and communication connected to the internet. This technology entails facility identification, construction of the network and attaching sensor devices and controls [9]. The introduction of IoT in healthcare building facilities for a smart healthcare building could be achieved via leveraging sensor devices, cloud computing, methods of connection, databases, internet protocols, and analytics as infrastructure, using different systems together [2]. IoT technologies and smart healthcare building facilities are used for different reasons, including the reduction in the maintenance costs of healthcare building facilities, reduction in operating costs of machinery and equipment in the healthcare services, enhancement of patient treatments through the reduction in diagnostic delays, increase in staff and patients comfortability, early detection of deterioration in the healthcare buildings, improvement in general safety for both patients and staff, provision of energy efficiency in the healthcare buildings, and general improvement in the profitability [9].

IoT enables the automation and detection of various defects in healthcare buildings for prompt measurement and remedial services. IoT-based vital measurement sensor devices have been developed and attached to building facilities to measure any identified defects [9]. Kang et al. [20] stated that barcodes technologies, RFID technologies, fingerprint/iris/face recognition technologies, and ultrasound-based recognition technologies are used in smart healthcare buildings to deliver better and faster services. The most commonly used IoT-based system is RFID technology, which is used for healthcare building facility management and medical services [21]. 

**Figure 1:**
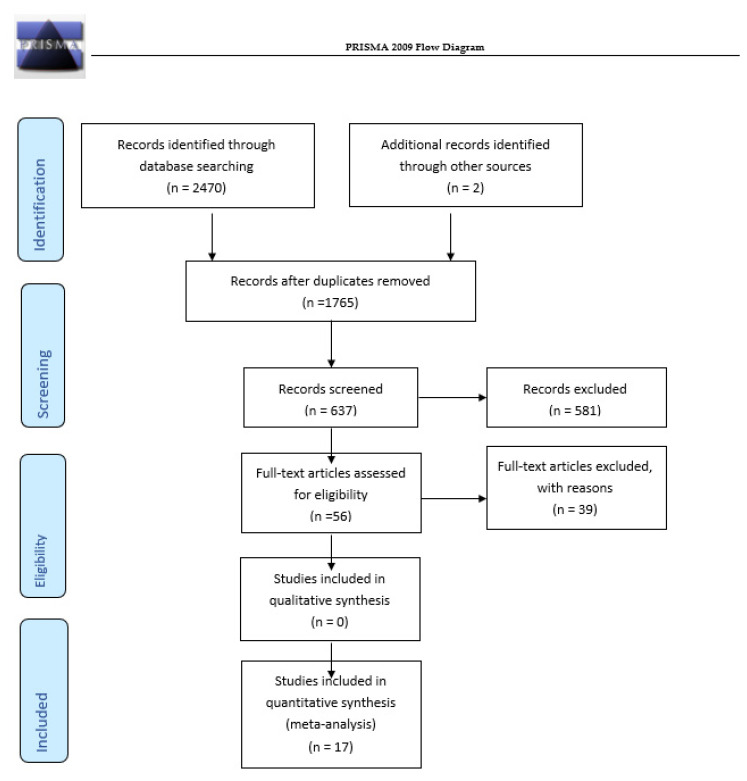
Flowchart for the systematic review procedure.

The flowchart for the systematic literature review procedure is presented in Figure 1. The initial search yielded 2470 studies. After eliminating duplicates, 1765 remained. Following an initial check of the titles and abstracts, 581 studies were removed, as they did not meet the inclusion criteria, leaving 56 studies to be read thoroughly. Finally, 17 studies met all of the inclusion criteria and formed the basis for the review.

- Authors, year of publication and country of data collection.
- Area of study, i.e., smart building infrastructure in relation to the healthcare building infrastructure.
- Applications of technologies such as IoT towards achieving smart building infrastructural facilities.
- Measured variables used in the studies and their outcomes.
- The main findings of the studies and the implications of the results.

## 2. Materials and Methods

This study reviewed papers exploring the relationship between IoT and smart building infrastructure applications. In addition, this study aimed to assess the impact of different IoT services towards achieving smart healthcare building facilities. Subsequently, based on the literature, a conceptual framework that binds the relationship between different IoT services and smart primary healthcare building facilities was developed. Hence, the questionnaire used to collate the data for this study had four (4) study constructs. The constructs consisted of three (3) constructs of IoT services and one (1) construct of the factors of the smart primary healthcare building facility. Hence, this study adopted a quantitative design [22]. The area of this study is smart healthcare building facilities. 

A randomly selected group of 750 project managers and healthcare practitioners working within the primary healthcare sector in Gauteng province, South Africa, were asked to complete the administered questionnaires. These questionnaires were administered to the respondents through WhatsApp. About 420 questionnaires were retrieved and 400 were used, while 20 were rejected because of inconsistencies in the responses. The analysis represents 56% and 53% return and response rates, respectively [23].

The main instrument for this research was an online administered questionnaire (Supplementary Materials), and the questionnaire contains only closed-ended questions [24]. The adapted questions used in the questionnaire were captured by four (4) study constructs of smart healthcare building facilities (SMAHEAL)-related factors, used as a dependent variable, and IoT location recognition and tracking services (IoT-LORE), IoT high-speed communication network-based services (IoT-HISB), and IoT-based services (IoT-BAS) were the independent variables, respectively. All the constructs used were measured using a 5-point Likert scale through the development of a model. Partial least squares structural equation modelling (PLS-SEM) was used for data analysis.

Hair et al. [25] suggested that the PLS-SEM software (WarpPLS 8.0, ScriptWarp Systems, Laredo, TX, USA.) can be used to enable the development of theories through the establishment of causal relationships among the study constructs. Contrarily, covariance-based structural equation modelling (CB-SEM) is commonly used to confirm relationships and theories among constructs [25]. Furthermore, PLS-SEM software is used for data analysis due to its high predictive capability, and it is used to assess the validity of the measured constructs [25]. The measurement model of this study was shown in Figure 1. This indicated the number of items in each study construct. Similarly, Table 1 shows the sources from which the items of each construct were adapted.

### Theoretical Frameworks

This study adopted two (2) theories: the scientific management theory and Schumpeter’s innovation theory. The two (2) theories tend to explain the relationship between the application and use of technology in minimizing the causal effect of variables., i.e., the application of technology such as IoT services to achieve enhanced building facilities services at the primary healthcare units for healthcare delivery services.

The scientific management theory was developed by Frederick Taylor [26] and suggests that scientific and technological methods should be used to perform tasks in the workplace, as opposed to leaders relying on their judgments or the personal discretion of team members. This theory was adopted because it reveals that scientific and technological methods, such as the application of IoT services towards enhancing smart building facility services, would result in the most productive workplace.

While Schumpeter’s innovation theory [27] is in line with other business investment theories, which assert that the change in business investment accompanied by monetary expansion is the major factor behind business improvement. Schumpeter’s theory posits that business innovation is the major reason for enhancing service delivery, therefore improving the productivity in investments and business success. These two theories explain the relationship between the study constructs. Accordingly, the theories advocate for the adoption of technology to enhance service delivery in healthcare building facilities. 

The research constructs cover the IoT service factors that enable the achievement of smart primary healthcare building facility services. The IoT services comprise three (3) constructs: IoT-LORE, IoT-HIBS, and IoT-BAS, while the dependent variable is a smart primary healthcare building facility (SMAHEAL). All four constructs, i.e., dependent and independent variables were rated using a 5-point Likert scale. This scale concerns uni-dimensionality and is the most common scaling process used in engineering management research [28]. The operationalization of the independent variables was achieved by rating the scales from a very low to a very high impact rating. In contrast, the dependent variable was rated from a very low-level to a very high-level rating for the healthcare facility. The operationalization process was adapted from Gambo and Musonda’s [29] studies. The data were collected by online administered questionnaires, which were retrieved and analysed using PLS-SEM algorithms.

**Figure 2:**
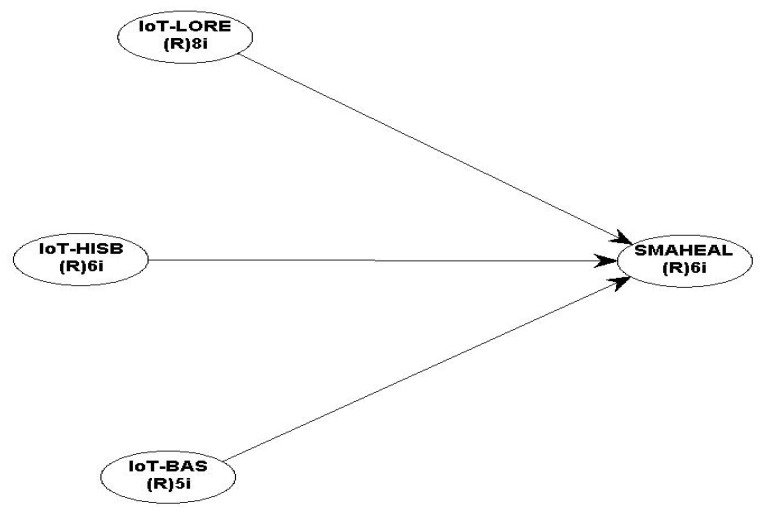
Measurement model.

According to the measurement model presented in Figure 2. The following directional alternate hypotheses were developed:

**H_A1_:**  *There is a significant positive impact between the application of IoT location recognition and tracking services and the achievement of smart primary healthcare building facilities.*

**H_A2_:**  *There is a significant positive impact between the application of IoT high-speed communication network-based services and the achievement of smart primary healthcare building facilities.*

**H_A3_:**  *There is a significant positive impact between the application of IoT-based services and the achievement of smart primary healthcare building facilities.*

## 3. Results 

### 3.1. Respondents’ Demographic Information

Table 2 indicates the demography of the respondent groups. The respondent group comprises experienced project managers and healthcare practitioners working within the public sector in Gauteng, South Africa. The statistics indicate that about 51.75% of the respondents were project managers, while the remaining 48.27% were primary healthcare practitioners. The results further show that 10.00% held PhDs, 40.25% held M.Sc., and the remaining 49.75% held BSc. The average working experience of the respondents was approximately thirteen (13) years, indicating that the respondents were experienced enough with primary healthcare building provision. 

### 3.2. Indicators of the Model Fits

Past studies focused on some essential sets of guidelines that use confirmatory factor analysis (CFA) as the primary statistical analysis technique. The statistical indicators include the following: alike information criteria and chi-square (χ^2^), parsimonious fit, comparative fit, goodness-of-fit index, standardized root mean square residual (SRMR), root means square error (RMSE), and Bentler–Bonett or Normed fit index [25].

Moreover, Kock [24] differentiated that there is a philosophically forthright distinction between CB-SEM and PLS-SEM. This study used PLS-SEM based on the objective of the study. The research objectives of this study are intended for theory development and testing. In this case, the best method to use is PLS-SEM. Therefore, the research objectives are intended for the prediction and development of theories. Therefore, the present study entails theory development and prediction. Conceptually, PLS-SEM is similar to a multiple regression analysis of data.

Based on the interpretation of the model fits, when the target of the research is to test the developed hypotheses, if the position of each arrow in the conceptual model signifies a hypothesis, then the fit indices are less important. However, if the intention is to assess how the collated data fit into the developed model, then the indices of the model fits are vital indicators of the quality of the model [24].

However, the Warp PLS-SEM algorithms indicated the following indices that compare the indicators of the correlation matrices and include: the standardised mean absolute residual (SMAR), standardised root means squared residual (SRMR), standardised chi-square (SChS), standardised threshold difference count ratio (STDCR), and standardised threshold difference sum ratio (STDSR). However, some classic model fits and the explanation of their indices depends on the aim of the study. Subsequently, the indices denote the fits between the empirical indicator correlation matrices and implied model. The indices become more expressive when the aim is to assess whether the model fits better with the collated data than the other, mainly when used together with the common indices [24].

An analysis of the model fits indicated the following statistics: the average path coefficient (APC) was 0.308 with a significant *p*-value < 0.001; the average R-squared had a statistics value of 0.635 with a *p*-value < 0.001; and the average adjusted R-squared (AARS) had a statistical value of 0.632 with a *p*-value < 0.001. The statistics of average block VIF (AVIF) was 1.686, which is regarded as acceptable since it is ≤5. Ideally, this value should be ≤3.3 for the AVIF in this model is regarded as ideal. The average full collinearity VIF (AFVIF) had a statistical value of 2.090, regarded as acceptable since it is ≤5. Ideally, this value should be ≤3.3 for the AFVIF in this model to be regarded as ideal. The VIF measures are used when indicators are formative. 

The model fits also indicated that the Tenenhaus GoF (GoF) had a statistical index of 0.522, regarded as small if it is ≥0.1, medium if it is ≥0.25, and large if it is ≥0.36. Therefore, the GoF value in this study was regarded as significant, and the GoF is the geometric average of the commonality. The average R^2^ of endogenous variables signifies the index for validating the PLS model. Generally, it is a compromise between the performance of the instrument and the developed structural model. 

The results further indicate that the Simpson’s paradox ratio (SPR) had a value of 1.000, regarded as acceptable if greater than or equal to 0.7 and ideal if it is 1.00. Therefore, the SPR in this model is ideal. The R-squared contribution ratio (RSCR) has a statistic of 1.000, regarded as acceptable if it is ≥0.9. Ideally, this value should be =1; therefore, the RSCR of this model was regarded as an ideal. The statistical suppression ratio (SSR) had a statistical value of 1.000, which was considered acceptable if greater than or equal to 0.7; therefore, it is acceptable in this model. The nonlinear bivariate causality direction ratio (NLBCDR) had a statistical value of 1.000, regarded as acceptable if it is greater or equal to 0.7; therefore, it is seen as acceptable. Hence, based on the above statistical indices, this model is considered to have good fit indices [24]. 

### 3.3. Measurement Model

Table 2 indicates an assessment of the model, which commonly follows two stages: the evaluation of the measurement and the structural model parameters [25]. The measurement model estimates determine the instrument’s validity and reliability, including the correlations among the indicators. Thus, the model has four (4) constructs: SMAHEAL, IoT-LORE, IoT-HIBS, and IoT-BAS. 

The measurement model appraises the model’s reliability and validity, which have the following two criteria: composite reliability (CR) and the average variance extracted (AVE) [25]. Moreover, earlier studies involved internal consistency to test the reliability of the instrument. According to Sijtsma [30], Cronbach’s alpha (α) tests the consistencies of the questionnaire and the level of the random error. Using Cronbach’s alpha allows for a quick detection of negative factors and their removal, with positive values ranging from 0.0 to 1.0 [25]. The minimum suitable value of Cronbach’s alpha is 0.6 [31]. Once an item is used as a scale, the item is required to be within the benchmark (0.60 and above) for the values of the reliability indicators. Ho [31] stated that the reliability of an instrument is defined as its capability to quantify the circumstance it is intended to accurately measure. 

Construct reliability refers to a test of consistency. The significance of reliability is a precondition for the validity of the study instrument. The consistency test encompasses an item analysis, split-half technique, and Cronbach’s alpha method. The main inadequacy of Cronbach’s alpha test is that it underestimates the consistency of a variable with smaller sample sizes of less than a hundred (100) units. Nonetheless, a construct with a large sample size of more than a hundred (100) units is considered exceptional. Cronbach’s alpha is employed to examine the internal consistency of variables [25]. Moreover, Ho [31] suggested Cronbach’s alpha test to be dependable in comparison to other tests. The Cronbach’s alpha test delivers an exclusive estimation of a scale’s internal consistency.

Consequently, study indicators and construct reliability are examined to evaluate the consistencies of the measurement model. Two tests are used to assess the reliability of the construct, i.e., the composite reliability (CR) and Cronbach’s alpha tests. Hair et al. [25] suggested using CR for PLS-SEM software. On the other hand, validity was assessed by double-checking the loading of the indicator on its related construct, and the loading should be 0.70 and higher before accepting the validity of the indicator [25]. 

Table 3 shows the results of the measurement model. The results indicate a high validity and internal consistency among the indicators in the model. All the factor loadings were greater than the benchmark of 0.70, whereas the CR and Cronbach’s alpha ranged from 0.702 to 0.846 and 0.718 to 0.775, respectively. This shows that all the indicators and the reliabilities were adequate. The discriminant and convergent validities were also considered in the validation of the measurement model. The AVE values of the constructs should be 0.50 and higher for a recognized convergent validity test [25]. The AVE is only used for models with reflective indicator variables. The AVE assesses the entire variance of a construct over its indicators [31]. The AVE figures of this model oscillated from 0.500 to 0.585, indicating that they were within the acceptable benchmark of 0.500, thus indicating the acceptability of the convergent validity of the instrument.

Table 4 shows the measurement model’s discriminant validity. The validity is the degree to which the construct is distinguished from other constructs in the model. This is attained by double-checking the AVE of the construct, which must be greater than the largest squared correlation of the construct. Alternatively, the loading of the construct must be greater than the other constructs in the model [25]. These results show that the square root of AVE for the construct and its relationship with another construct is an acceptable discriminant validity. Based on the above results, the questionnaire was regarded as valid and reliable for its intended purpose.

### 3.4. The Measures of the Path Coefficients of the Developed Model

**Figure 3:**
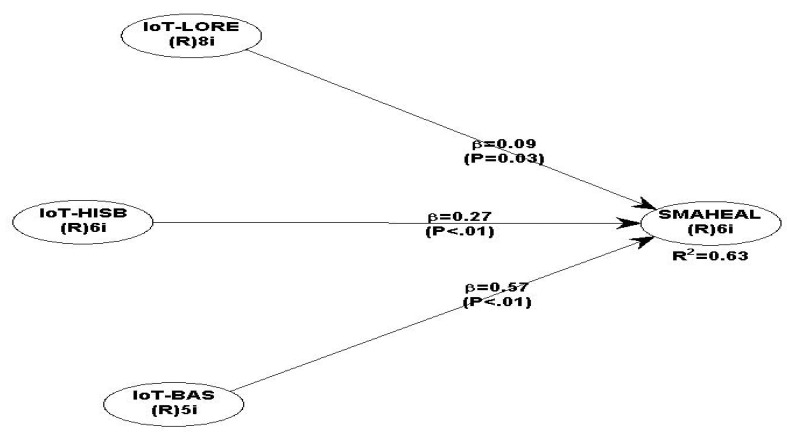
Structural model of the constructs.

Figure 3 shows the coefficient of determination (R^2^) as the measure of the endogenous variables and the path coefficients of the developed model. This is appraised as a component of an initial evaluation of the structural relationships, i.e., the inner developed model from the conceptual/hypothetical framework of this study [25]. Thus, Chin [32] suggested that a R^2^ value of 0.67 was substantial, while 0.33 was moderate, and a value of 0.19 was weak. In the current study, the value of R^2^ was determined to be 0.635, indicating a significant and moderate relationship between the criterion (IoT services) and predictor variables (SMAHEAL). The path coefficients between IoT-LORE, IoT-HABS, IoT-BAS and the dependent variable SMAHEAL were 0.090, 0.265, and 0.570, which were all significant at a P_0.05_ level of significance, respectively.

Table 5 shows the effect significance (f^2^) of an independent variable on dependent variables forecasted by the path coefficients. The effect was described as either low for the value of 0.02, moderate for the value of 0.15, or high for the value of 0.35 [33]. The value of f^2^ shows that the impact of a specific construct on the dependent variable is considerable [32]. The values of f^2^ between IoT-LORE, IoT-HIBS, IoT-BAS, and the dependent variable SMAHEAL were 0.018, 0.178, and 0.439, indicating low, moderate, and high effects, respectively. Similarly, the predictive capability of the endogenous constructs in the relationship was assessed using Stone–Geisser’s cross-validated redundancy (Q^2^). The predictive ability of the relationship was 0.635, based on the generated value in the model Q^2^ [24]. However, Hair et al. [25] found that Q^2^ values show the predictive significance as either weak for the value of 0.02, moderate for the value of 0.15, and strong for the value of 0.35. Consequently, this model shows a robust predictive significance because Q^2^ > 0: 0.635 to be precise. Therefore, the path model’s predictive significance to the construct was determined to be strong. This implies that the predictors (IoT-LORE, IoT-HIBS, IoT-BAS) predicted about a 64% achievement of SMAHEAL whenever adopted. 

### 3.5. Discussion of Results

This study assessed the impact of IoT services on achieving smart primary healthcare building facilities. This study theorized four (4) constructs: SMAHEAL (dependent variable), IoT-LORE, IoT-HIBS, and IoT-BAS. The constructs were adapted from previous studies on IoT and smart healthcare buildings. 

The study measurement model indicates that the research instrument was highly reliable and valid for the intended purpose, therefore demonstrating the reliability and validity of the results. This study found significant variable impacts between the IoT services (IoT-LORE, IoT-HIBS, and the IoT-BAS) and SMAHEAL. The impact of the SMAHEAL and the IoT-LORE equally indicated that a significant but low impact (0.018) existed between the two constructs [33]. The findings support the hypothesis that a significant positive impact existed between the application of IoT location recognition and tracking services and the achievement of smart primary healthcare building facility services. These findings agree with those of Jia et al. [6], who argued for the need of adopting IoT for the development of smart buildings. This also indicates a small impact between the IoT location recognition and tracking services for the smart infrastructure. However, the study contradicts the findings of Bagheri and Movahed [34] on the effect of IoT on the education business model, where a moderate relationship was found. This is because the study considered general learning and educational environments without infrastructure facilities.

Similarly, the results show that there is a significant and moderate impact (0.178) between IoT-HIBS and SMAHEAL [33], which supports the hypothesis that there is a significant positive impact between the application of IoT high-speed communication network-based services and the achievement of smart primary healthcare building facility services. This is in line with Ramesh et al. [35], who evaluated the achievement of sustainability through smart city applications, protocols, systems and solutions using IoT and wireless sensor networks. Their research found a moderate relationship between sustainable smart cities and high-speed communication network protocols. In addition, the current study builds on the study by Ahad et al. [36], which assessed the technological trend toward 5G networks for smart healthcare using IoT services, in which one indicator of IoT-HIBS, 5G networks, was considered.

Lastly, the results also indicated a high impact (0.439) between IoT-BAS and SMAHEAL [33]. The findings supported the hypothesis that there is a significant positive relationship between the application of IoT-based services and the achievement of smart primary healthcare building facility services. This is in line with the study of Lawal and Rafsanjani [37], which assessed the trends, benefits, risks, and challenges of IoT implementation in residential and commercial buildings and concluded that IoT-based services significantly improve the smartness of building infrastructure. Contrarily, the results of this study contrast with those of Jia et al. [6], who studied the adoption of IoT for the development of smart buildings and found a weak relationship between IoT-based services and the development of smart cities.

## 4. Conclusions

This study aimed to assess the impact of IoT services (IoT-LORE, IoT-HIBS, and IoT-BAS) on achieving smart primary healthcare building facility services (SMAHEAL). With a view to improve the delivery of primary healthcare building facility services in developing countries. The study identified three (3) basic constructs of IoT services comprising the application of IoT location recognition and tracking services (IoT-LORE), the application of IoT high-speed communication network-based services (IoT-HIBS), and the application of IoT-based services (IoT-BAS), which are designed to help achieve smart primary healthcare building facility services for rapid healthcare delivery in rural areas of developing countries. 

This study found that there are variable effects between the three (3) IoT services and achieving smart primary healthcare building facility services. The results indicate low-, moderate-, and high-impact changes for IoT-LORE, IoT-HIBS, and IoT-BAS with SMAHEAL, respectively. The findings implied that positive variable degrees of impacts existed between the exogenous and the endogenous variables and that the application of IoT services positively and variably aided the achievement of smart primary healthcare building facility services, which in turn could enhance primary healthcare service delivery in developing countries. 

Therefore, this study recommends adopting IoT services towards achieving primary healthcare services in rural areas of South Africa and other developing countries with similar challenges regarding primary healthcare building facility services such as South Africa. Consequently, this study recommends further research on the adoption of IoT services for achieving smart primary healthcare services to fully realize the potential that these technologies can offer for rural communities.

### Limitations

- This paper used a Likert scale approach for it analysis, relying on the personal experiences of respondents in primary healthcare building delivery rather than actual surveys of the facilities in the buildings, and participants may be reluctant to express their true views due to social expectations and moral pressures; therefore, findings should be treated with caution.
- The survey design used a cross-sectional approach, so it is only able to capture experience, beliefs and behavioural intentions at a single point in time. Given that experience, beliefs and behavioural intentions change over time, future research could explore this from a para-experimental perspective or use a longitudinal approach or time series data for follow-up studies.
- This study is based on the assessment of the impact of IoT on smart primary healthcare building facilities, but IoT is not the only technology used for the assessing smartness of infrastructure facilities. Other technologies such as artificial intelligence (AI) and virtual and augmented realities should also be applied in further studies to consider the effects of different technologies on achieving smartness in building facilities.

## Figures and Tables

**Table 1:** The sources of items in the study constructs.

	Khan and Salah (2018) [11]	Wassie et al., (2022) [2]	Kwon et al., (2022) [9]	Birje and Hanji (2020) [16]	Verdejo et al., (2021) [15]	Schuchat et al., (2020) [17]	Zhao and Jiang (2018) [7]	Yan (2019) [19]	Singh and Mahapatra (2020) [18]	Wassie B. et al., (2022) [2]	de la Torre, D. (2019) [21]	Kwon et al., (2022) [9]	Kang (2019) [20]
**SMAHEAL**													
Interoperability of the building facilities	√		√										
Mobile integrated solution		√	√										
Digitisation of information		√											
A unified system of communication system		√	√										
Stable core infrastructure facilities	√		√										
System automation	√		√										
**IoT-LORETS**													
Beacons technologies		√		√	√	√							
Bluetooth technologies				√									
Wi-Fi technologies				√	√								
Zigbee technologies				√		√							
RFID technologies			√										
GPS and A-GPS Technologies			√		√								
Barcodes and QR codes			√			√							
Ultra-wideband communication			√		√	√							
**IoT-HISBAS**													
Integrating IoT into 5G and B5G high-speed communication								√	√				
Wi-Fi 6						√			√				
OFDMA technology					√			√					
infusion pump							√		√				
Sensors and wearables for IoT-based wireless health						√			√				
Facility-to-facility connectivity with high mobility					√		√	√					
**IoT-BAS**													
Facilities identification										√		√	√
Network construction										√	√	√	
Sensor attachment										√			√
**Sensor control**											√	√	
Cloud computing and analytics										√	√		√

**Table 2:** Information on respondents’ demography.

Project Managers	No.	%	Cumulative %	
Project Managers	207	51.75	51.75	
Healthcare Practitioners	193	48.25	100	
Total	400	100		
**Educational Qualifications**				
PhD	40	10.00	10.00	
MSc	161	40.25	50.25	
BSc	199	49.75	100	
**Total**	400	100		
**Experience of Respondents (Year)**				
**Years**	**Mid Value (x)**	**Frequency (f)**	**% of Frequency**	**Fx**
**5–10**	7.5	85	21.25	637.50
**10–15**	12.5	109	27.25	1362.50
**15 and above**	15.0	206	51.50	3090.00
**Total**		400	100	5090.00

Calculated mean (average) years of working experience of the respondents Σfx/Σf = 5090.00/400 = 12.73 ≈ 13 mean years of working experience.

**Table 3:** Assessment of the study measurement.

Construct	Indicators	Indicator Loading	CR	Cronbach’s α	AVE
SMAHEAL	SMAHEAL1	0.832	0.846	0.775	0.500
	SMAHEAL2	0.824			
	SMAHEAL3	0.754			
	SMAHEAL4	0.856			
	SMAHEAL5	0.275			
	SMAHEAL6	0.719			
IoT-LORE	IoT-LORE1	0.793	0.702	0.730	0.524
	IoT-LORE2	0.870			
	IoT-LORE3	0.873			
	IoT-LORE4	0.768			
	IoT-LORE5	0.784			
	IoT-LORE6	0.774			
	IoT-LORE7	0.726			
	IoT-LORE8	0.733			
IoT-HISB	IoT-HISB1	0.759	0.753	0.718	0.585
	IoT-HISB2	0.768			
	IoT-HISB3	0.768			
	IoT-HISB4	0.761			
	IoT-HISB5	0.730			
	IoT-HISB6	0.721			
IoT-BAS	IoT-BAS1	0.769	0.837	0.756	0.508
	IoT-BAS2	0.795			
	IoT-BAS3	0.725			
	IoT-BAS4	0.795			
	IoT-BAS5	0.770			

Note: α-alpha; CR—composite reliability; AVE—average variance extracted.

**Table 4:** Results of discriminant validity.

	SMAHEAL	IoT-LORE	IoT-HISB	IoT-BAS
SMAHEAL	0.707			
IoT-LORE	0.133	0.570		
IoT-HISB	0.656	0.018	0.620	
IoT-BAS	0.701	0.098	0.621	0.712

Note: Discriminant validity showing AVE.

**Table 5:** Testing of the study hypotheses.

Hypotheses	Path Coefficient	*p*-Value	Effect Size (f^2^)	Stone–Geisser’s Q^2^	R^2^	Supported
IoT-LORE→SMAHEAL	0.090	0.035	0.018	0.635	0.635	Yes
IoT-HIBS→SMAHEAL	0.265	<0.001	0.178			Yes
IoT-BAS→SMAHEAL	0.570	<0.001	0.439			Yes

Note: Level of significance (*p*) ≤ 0.05; Q^2^—cross-validated redundancy.

## Data Availability

Not applicable.

## Supplementary Materials

The following supporting information can be downloaded at: https://www.mdpi.com/article/10.3390/ijerph191811147/s1, Questionnaire on: Impact of IoT Towards Achieving Smart Primary Healthcare Building Facilities in Gauteng, South Africa.
