# Supplementary material for: Impact of IoT on Achieving Smart Primary Healthcare Building Facilities in Gauteng, South Africa
Source: Int J Environ Res Public Health. 2022 Sep 6;19(18):11147. doi: 10.3390/ijerph191811147 (PMC9516893; doi:10.3390/ijerph191811147)
Supplement: Supplementary file 1 [file ijerph-19-11147-s001.zip › ijerph-1830371-supplementary.pdf]

Introduction to Respondents

CARINBE

<u>**QUESTIONNAIRE ON:**</u>

Impact of IoT Towards Achieving Smart Primary Healthcare Building Facilities in Gauteng, South Africa

Dear Respondent

This questionnaire represents a part of a study which aimed at assessing the impact of IoT services towards achieving smart primary healthcare building facilities in the rural area of South Africa with a view to enhance primary healthcare delivery. The output of this research work is expected to help in improving primary healthcare building facility services in rural communities of South Africa. This research work will also provides an atmosphere of building connections, techno-orientations among project managers and healthcare practitioners in South Africa.

The objectives of the research work are to explore the major IoT technologies fostering smart primary healthcare buildings facility services and also to assess the impact of the IoT technologies services in achieving smart primary healthcare buildings in Gauteng, South Africa.

The information supplied by you will be collated and analyzed together with that of other respondents. This will constitute your valuable and immeasurable contributions to the success of this work. Please note that the answers provided by you will be kept confidential as special precautions have been taken in this regard. It will be greatly appreciated if you could kindly respond to all questions and submitted the questionnaire online via WhatsApp. I sincerely appreciate your valuable time and other contributions devoted to foster research in the field of smart infrastructure facilities.

Thank you!

Structured Research Questionnaires

Part A: Section 1: General Information ON Respondent

Please rate the appropriate option that best describe yourself in primary healthcare building facilities delivery. Please do not thick more than one option.

Q1. Which of the followings best describes your position in primary healthcare delivery services?

Project Manager [ ] Healthcare Practitioner [ ].

Q2. Which of the followings described your level of education qualification?

PhD [ ] Msc [ ] Bsc [ ]

Q3. Which of the followings described your years of services in primary healthcare?

5–10 years [ ] 10–15 years [ ] Above 15 years [ ]

Part B: Section 2: Information about Technologies Influencing Smart Healthcare Building Facilities

Please rate the appropriate option that best describe the technologies influencing smart healthcare building facilities in Gauteng province, South Africa. Please do not rate more than one option.

Technologies Influencing Smart Primary Healthcare Building Facilities

Rate the level of smartness of primary healthcare building facilities in the Gauteng Province of South Africa based on the following questions

Very Low Level of Smartness	Low Level of Smartness	Moderate Level of Smartness	High Level of Smartness	Very High Level of Smartness
1	2	3	4	5

SMART TECHNOLOGIES:

NO	QUESTIONS/ STATEMENTS	1	2	3	4	5
1	Ease of interoperability of the building facilities within the healthcare building					
2	Presence of mobile integrated solution in the healthcare building					
3	Digitization of information in the healthcare building					
4	A unified system of communication system available in the healthcare building					
5	Presence of stable core infrastructure facilities in the healthcare building					
6	Application of system automation in the healthcare building					

Part C: Section 3: Information about Iot Services in Healthcare Buildings

Rate the impact of the IoT services towards achieving smart primary healthcare buildings below

Very Low Impact	Low Impac	Moderately Impact	High Impact	Very High Impact
1	2	3	4	5

IOT LOCATION RECOGNITION AND TRACKING SERVICES:

NO	QUESTIONS/ STATEMENTS	1	2	3	4	5
1	Availability of beacons technologies in the healthcare building					
2	Presence of Bluetooth technologies in the healthcare building					
3	Existence of Wi-Fi Technologies in the healthcare building					
4	Presence of Zigbee technologies in the healthcare building					
5	Availability of RFID technologies in the healthcare building					
6	Presence of GPS and A-GPS technologies in the healthcare building					
7	Uses of Barcodes and QR codes in the healthcare building					
8	Presence of ultra-wideband communication in the healthcare building					

IOT HIGH-SPEED COMMUNICATION NETWORK-BASED SERVICES:

NO	QUESTIONS/ STATEMENTS	1	2	3	4	5
1	Availability of integrated IoT into 5G and B5G high-speed communication in the healthcare building					
2	Presence of Wi-Fi 6 in the healthcare building					

3	Uses OFDMA technology in the healthcare building	
4	Uses of infusion pump in the healthcare building	
5	Availability of sensors and wearables for IoT-based wireless health in the healthcare building	
6	Uses of facility-to-facility connectivity with high mobility in the healthcare building	

IOT-BASED SERVICES:

NO	QUESTIONS/STATEMENTS	1	2	3	4	5
1	Availability of facilities identification device in the healthcare building					
2	Availability of network construction device in the healthcare building					
3	Presence of sensor attachment devices in the healthcare building					
4	Presence of sensor control devices in the healthcare building					
5	Uses of cloud computing and analytics in the healthcare building					
